# Supplementary material for: Intestinal Anti-Inflammatory Effect of a Peptide Derived from Gastrointestinal Digestion of Buffalo (Bubalus bubalis) Mozzarella Cheese
Source: Nutrients. 2019 Mar 13;11(3):610. doi: 10.3390/nu11030610 (PMC6471453; doi:10.3390/nu11030610)

Supplementary files

MBCP reduces intestinal permeability in DNBS-induced colitis in mice. Effect of MBCP (100 mg/kg, by oral gavage) on serum FITC–dextran concentration, a measure of intestinal permeability. FITC (600 mg/kg) was detected in the serum 24h after its administration and three days after the induction of colitis by DNBS (150 mg/kg). All data are represented as mean ± SEM of 6 mice for each experimental group. Statistical significance was calculated using one-way ANOVA test. #p<0.001 vs control, **p<0.01 vs DNBS alone.

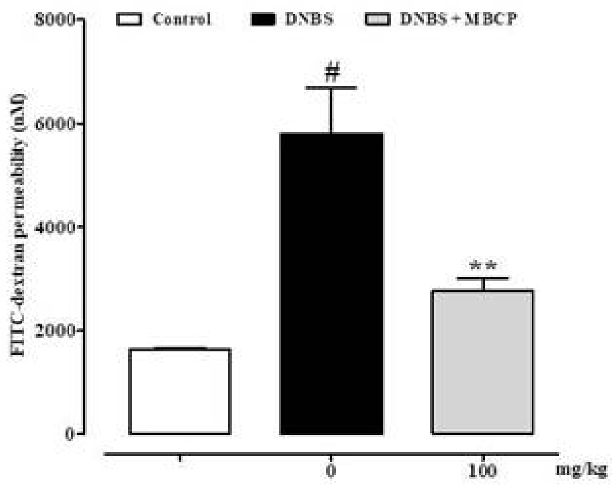

Supplement: Supplementary file 1 [file nutrients-11-00610-s001.pdf]
